# Relationship between Changes in Condylar Morphology and Masticatory Muscle Volume after Skeletal Class II Surgery

**DOI:** 10.3390/jcm12144875

**Published:** 2023-07-24

**Authors:** Bunpout Lekroengsin, Chie Tachiki, Takashi Takaki, Yasushi Nishii

**Affiliations:** 1 Department of Orthodontics, Tokyo Dental College, Tokyo 101-0061, Japan; nishii@tdc.ac.jp; 2 Department of Oral and Maxillofacial Surgery, Tokyo Dental College, Tokyo 101-0061, Japan; ttakaki@tdc.ac.jp

**Keywords:** mandibular condyle, class II, orthognathic surgery, masticatory muscle

## Abstract

The treatment of dentofacial deformities generally includes orthognathic surgery in which mandibular condyle changes following surgery are a common cause of relapse. This study investigated the changes in the mandibular condyle and related muscles to identify the factors that affected the changes in the mandibular condyle after orthognathic surgery in skeletal class II patients. This research studied 60 joints in 30 patients with skeletal class II dentofacial deformities who received surgical orthodontic treatment, including bilateral sagittal split ramus osteotomy, and underwent computed tomography before and after orthodontic treatment. The mandibular condyle, masseter, and medial pterygoid muscles were reconstructed and measured in 3D. Condylar positional and morphology changes, masseter and medial pterygoid muscle volume, temporomandibular joint (TMJ) pain, and distal segment movement were analyzed. The study observed that both the masseter and medial pterygoid muscle volumes decreased with statistical significance. The changes in the horizontal direction were positively correlated with the amount of movement. The findings indicated that mandibular condyle changes were significantly affected by the movement of the distal segment, the medial pterygoid muscle volume, and the direction of the distal segment, which influenced the treatment’s long-term stability after orthognathic surgery.

## 1. Introduction

**Figure 1:**
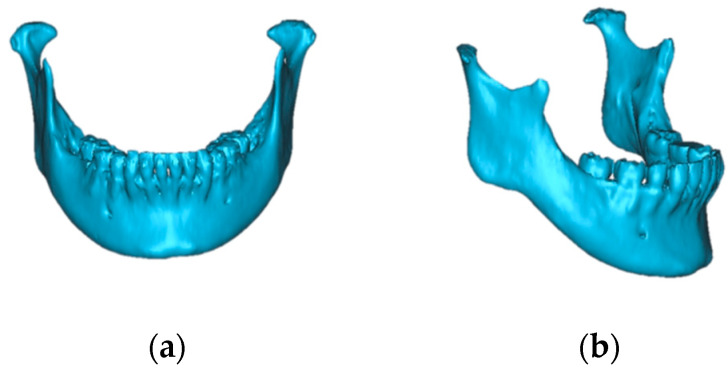
Changing condylar morphology following orthognathic surgery leading to postsurgical relapse: (**a**) the pre-treatment mandibular condyle; (**b**) the post-treatment mandibular condyle: degenerative changes.

Patients with dentofacial deformities frequently exhibit a variety of abnormalities that manifest in various aspects of their oral and facial structures. Orthognathic surgery is commonly used to correct the deformities. However, following orthognathic surgery, degenerative changes in the condylar morphology (Figure 1) influence relapses [1,2].

Many studies have assessed changes in the mandibular condyle position due to orthognathic surgery [3,4,5,6,7,8]. Some studies reported that orthognathic surgery could induce significant changes in maximum bite force levels and temporomandibular joint (TMJ) load, thus affecting condylar morphology [9,10,11]. Furthermore, surgical advancement may alter the biomechanics of the mandible, resulting in an improvement or deterioration in masticatory function [12]. The primary masticatory muscles—the temporalis, medial pterygoid, lateral pterygoid, and masseter—as well as the accessory muscles—the buccinator, suprahyoid muscles, and infrahyoid muscles—work together to produce mandibular movement [13]. The changes in the masticatory muscles are influenced by eating habits that could alter the stiffness of the muscles and are associated with masticatory muscle disorders (MMD), including chronic temporomandibular disorder (TMD) and disk displacement with reduction (DDR), even in myopic patients in which refractive errors are increased [14,15,16]. Regarding changes in the masticatory muscle, we aim to address the hypothesis of whether changes in masticatory muscle volume affect changes in condylar morphology after surgical orthodontic treatment. However, in skeletal class II dentofacial deformities, few studies have evaluated 3D changes in the position of the mandibular condyle and mandibular body segment related to condylar morphology.

Furthermore, no research has been conducted to investigate the association between changes in masticatory muscle volume and condylar morphology following surgical orthodontic treatment. In addition, there are few reports regarding the relationship between TMJ pain and surgical orthodontic treatment of skeletal class II dental and facial deformities; furthermore, some points addressed therein still need to be clarified [3]. Therefore, in the current study, factors affecting changes in condylar morphology following surgical orthodontic treatment in skeletal class II dentofacial deformity patients and their association, including TMJ pain, were investigated.

## 2. Materials and Methods

**Figure 2:**
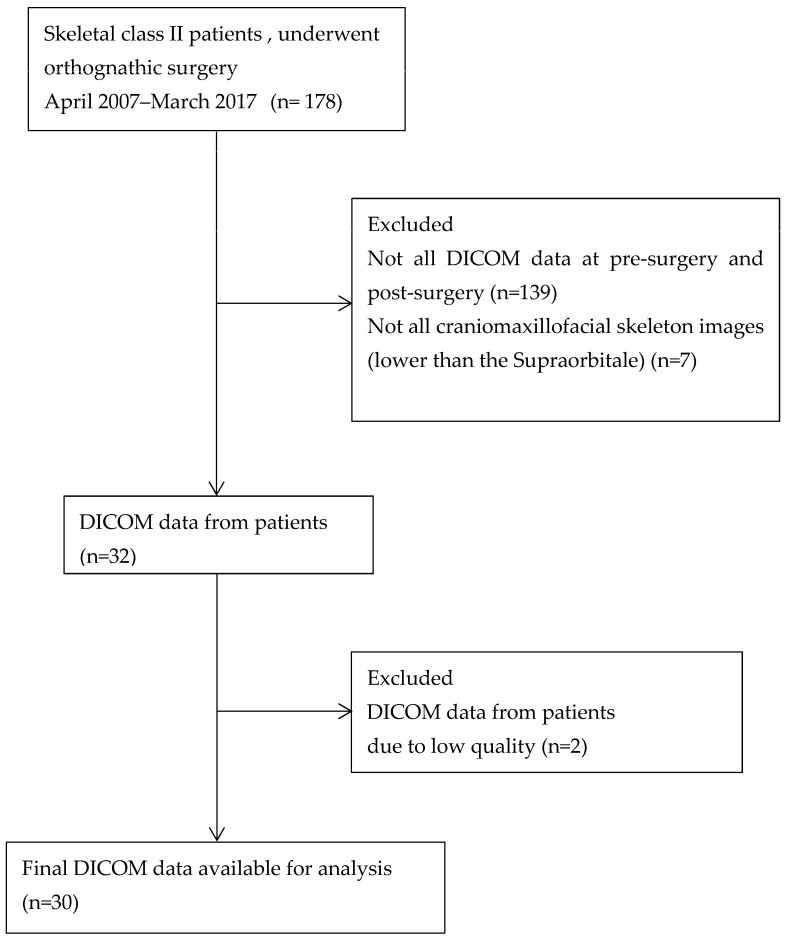
Study flow diagram.

This retrospective study examined patients at Tokyo Dental College Chiba Dental Center from April 2007 to March 2017. The inclusion criteria were as follows: (1) had been diagnosed with skeletal class II dentofacial deformities; (2) had undergone orthognathic surgery; and (3) had undergone CT (computed tomography) of the skull at the time of pre- and post- orthodontic treatment (1–2 years after orthognathic surgery). The exclusion criteria were (1) patients presenting with congenital disorders that give rise to orofacial and dental structural abnormalities and (2) individuals with a medical history involving tumors or trauma affecting the head and neck region. One hundred and seventy-eight CT images of pre- and post-treatment were collected in the first step. Of these, 146 images were excluded; specifically, 139 images lacked DICOM (Digital Imaging and Communication in Medicine) data pre- and post-treatment, and 7 lacked the required craniomaxillofacial images. Two of the remaining sources of DICOM data from patients were excluded due to inadequate quality. A final 30 patients achieved the criteria and were included in the current study (Figure 2). There were 4 men and 26 women among the patients. At the time of surgery, the average patient was 27.2 years old. A total of 14 patients had BSSRO (bilateral sagittal split ramus osteotomy) alone, and 16 had BSSRO combined with Le Fort I osteotomy, constituting a two-jaw surgery. Four specialized orthodontists diagnosed, planned, and conducted surgical orthodontic treatment using the same methodology. Two specialist oral surgeons performed all surgical procedures at the Department of Oral and Maxillofacial Surgery, Tokyo Dental College.

The skeletal pattern was evaluated pre-treatment using lateral cephalometric radiographs acquired at the initial exam. Regarding facial morphology in profile, Sassouni’s analysis [17,18] was used to classify the vertical skeletal pattern of the patients. Totals of 18 and 12 patients were classified as having long faces and average to short faces, respectively.

### 2.1. Orthognathic Surgery and Proximal Segment Repositioning

The orthognathic surgery methods used were bilateral sagittal split ramus osteotomy (BSSRO) alone or in combination with Le Fort I osteotomy as a two-jaw surgery. The condylar positioning method used for BSSRO involved marking the leading edge of the mandibular ramus at the height of the orthodontic archwire of the maxillary dental arch, followed by short lingual sagittal splitting [19]. Then, the angle of the mandible was pressed manually upward and towards the anterior border of the ramus posteriorly, and the condyle in the mandibular fossa was positioned following Arnett’s method [20]. Two specialist oral surgeons used a similar technique to carry out these operations.

### 2.2. Evaluation of Condylar Morphology Changes

CT scanners were used. The parameters were set to 120 kV, 160 mA, 23 cm field of view, and 0.6 mm slice thickness. Condylar morphology changes were assessed twice by a radiologist with more than five years of experience and a specialist orthodontist with ten years of experience via slices showing the condylar apical region of corrected sagittal and coronal sections. As in Yamada et al.’s prior study [21,22], reconstructed coronal and sagittal CT scanning was unable to detect typical sclerosis, concavities, or cysts in this study. As a result, diagnoses of normal, flattening, erosion, and osteophytes were adopted following Yamada et al.’s investigation [21,22].

### 2.3. Measurement of 3D Positional Changes in the Condyle

Simplant OMS software was used to reconstruct 3D images from CT DICOM data (Materialize Dental Co., Ltd., Leuven, Belgium). After orthognathic surgery, three-dimensional positional changes in the condyle (proximal segment) were measured [3].

**Figure 3:**
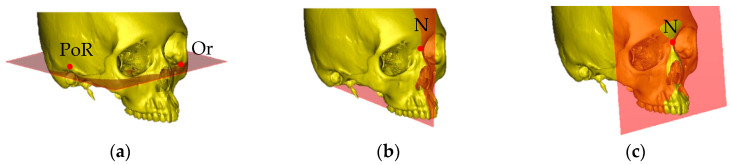
The 3D reference planes: (**a**) Frankfurt horizontal (FH) plane: the plane passing through Po (PoR, PoL) and the Or (OrR, OrL); (**b**) midsagittal reference (MSR) plane: the plane passing through N, S, and Ba; (**c**) nasion (N) plane: the plane passing through N and perpendicular to the Frankfurt horizontal plane.

According to the procedure described by Kim et al. [23], the following three reference planes in the maxilla were established: (a) the Frankfort horizontal (FH) plane; (b) the midsagittal reference (MSR) plane, which is the horizontal plane passing through the nasion (N), sella (S), and basion (Ba); and (c) the nasion (N) plane, which is a vertical plane passing through the nasion and perpendicular to the FH plane (Figure 3).

**Figure 4:**
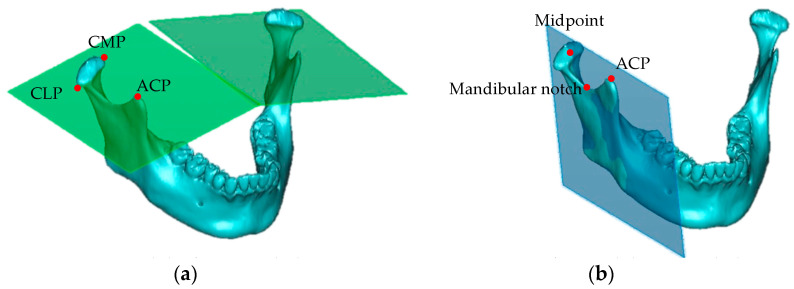
The 3D condyle measurement planes: (**a**) condylar horizontal plane: the plane passing through the condylar medial pole (CMP), the condylar lateral pole (CLP), and the apex of the coronoid process (ACP); (**b**) condylar vertical plane: the plane passing through the midpoint between the condylar medial and condylar lateral poles, the ACP, and the deepest point of the mandibular notch.

The following two planes in the mandible were measured to evaluate condylar positional changes: (a) the condylar horizontal plane, which passes through the medial and lateral poles of the condyle and the apex of the coronoid process and (b) the condylar vertical plane, which passes through the midpoint between the medial and lateral poles of the condyle, the apex of the coronoid process, and the deepest point of the mandibular notch (Figure 4).

**Figure 5:**
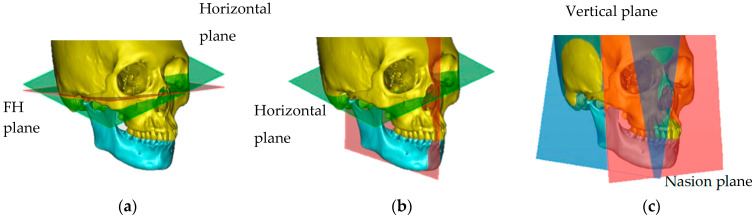
Measurement of the angle between the reference plane and the condyle measurement plane; (**a**) FH condyle angle (FCA): the angle between the FH plane and the condyle horizontal plane; (**b**) MSR condyle angle (MCA): the angle between the MSR plane and the condyle horizontal plane; (**c**) N condyle angle (NCA): the angle between the N plane and the condyle vertical plane.

The angles between the two condylar planes and the three reference planes were measured and averaged over three measurements. These were (a) the FH condyle angle (FCA), (b) the MSR condyle angle (MCA), and (c) the N condyle angle (NCA) (Figure 5).

### 2.4. Measurement of 3D Positional Changes in the Mandibular Body (Distal Segments)

After orthognathic surgery, changes in the movement and direction of 3D shifts in the distal portions were measured regarding the anterior margin of the mental foramen. The 3D coordination was obtained using 3D CT images of the skull at the initial exam and after treatment by matching at the basion and both anterior margins of the frontozygomatic suture via the interactive closest point method [24]. This result represented mandibular body displacement at the mental foramen’s left and right anterior edges, determined as the distance in three directions: *X*, *Y*, and *Z*.

### 2.5. Measurement of the Masseter and Medial Pterygoid Muscle Volume Changes

**Figure 6:**
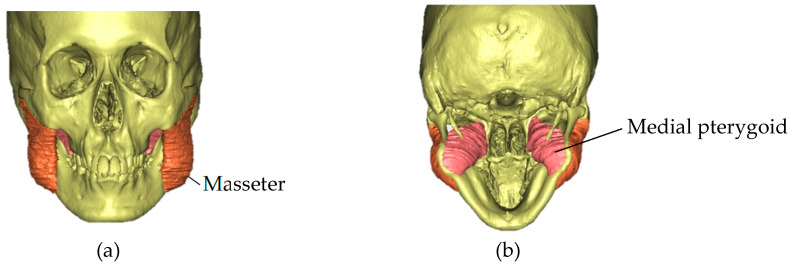
Reconstruction of the (**a**) masseter and (**b**) medial pterygoid muscles was performed; regarding the results of the reconstructed models, the SimPlant OMS Pro 14.0 software calculated the muscle volumes automatically.

Using DICOM files from CT, 3D images were reconstructed using Simplant OMS (Materialize Dental Co., Ltd., Leuven, Belgium). The 3D masseter and medial pterygoid muscle reconstructions were performed by sequential tracing of the muscle outline from the serial segmentation of the axial plane, utilizing CT [25]. Specialists in oral surgery and orthodontics confirmed the resulting traces. The muscle volumes were computed automatically by software based on the results of the reconstructed models (Figure 6). The masseter and medial pterygoid muscle volumes were compared pre- and post-treatment. The correlation coefficient according to the test retest reliability (in which the masseter muscle was traced to produce a 3D model three times in a row using the same method over a one-month period) was 0.91, and the intraclass correlation coefficient (ICC) was (1, 1) = 0.90, which is regarded as reliable and precise.

### 2.6. Statistical Analysis

Data were analyzed using SPSS 26.0 (IBM Corp., Armonk, NY, USA). The sample size estimation was based on previous studies by Dicker et al. [12,26]. The present study performed power analysis for non-parametric statistics. For a two-tail test, 25 individuals had an 85-percent power with an effect size of 0.6 and a significance level of 0.05. As a result, *n* = 30 was deemed suitable for the study. The Wilcoxon signed-rank test was performed to compare the volumes of the masticatory muscle pre- and post-treatment. Spearman rank correlation was used to evaluate correlation among variables, and logistic regression analysis was utilized to assess factors affecting the changes in condylar morphology. The differences were considered statistically significant when *p* < 0.05 and *p* < 0.01.

## 3. Results

Before orthognathic surgery, there was one joint with TMJ pain and 59 joints without. Postoperative pain relief was discovered in a patient with initial symptoms. However, postoperative TMJ pain was observed in two patients with no symptoms before surgery in this study.

### 3.1. The Changes in the Condylar Morphology after Orthognathic Surgery

Regarding the alterations of the mandibular condyle, which were classified according to conventional tomography applied by Yamada et al. [21,22] at initial treatment, 12 condyles (20%) were normal, and 48 condyles (80%) exhibited abnormalities. Of these 48 condyles, there were 38 cases of flattening, 5 cases of erosion, 1 case of osteophytes, and 4 cases had a combination of flattening and erosion. After completion of orthodontic treatment, the study found changes in the condylar morphology as 29 cases of flattening, 2 cases of erosion, 16 cases of a combination of flattening and erosion, 3 cases of a combination of flattening and osteophytes, and 6 cases of a combination of flattening, erosion, and osteophytes (Table 1).

### 3.2. Positional Changes in the Condyle and Mandibular Body in Three Dimensions

The analysis employed the values obtained by subtracting the postoperative measurements from the initial treatment measurements. The median positional changes were −2.56 [−0.51–(−4.36)]° for the FH condyle angle, 0.85 [3.56–(−1.83)]° for the MSR condyle angle, and −0.86 [1.28–(−1.46)]° for the N condyle angle. The median amount of movement was 5.67 (7.69–3.84) mm. The median positional changes in the *X*-, *Y*-, and *Z*-axis directions were 0.61 [1.61–(−0.44)] mm, 4.67 (7.18–2.85) mm, and 0.23 [1.99–(−1.30)] mm, respectively (Table 2).

### 3.3. The Changes in the Masseter and Medial Pterygoid Muscle Volume

Concerning the alterations in the masticatory muscle, at pre-treatment, the median volume was 16.96 (20.38–13.75) cm^3^ for the masseter muscle and 7.31(9.16–6.30) cm^3^ for the medial pterygoid muscle. Post-treatment, the median volume of the masseter and medial pterygoid muscles declined to 15.70 (17.76–12.82) cm^3^ and 6.56 (7.86–5.90) cm^3^, respectively. The masseter and medial pterygoid muscle volumes decreased significantly with p values less than 0.01 (Table 3).

### 3.4. Correlations between Distance and Direction of Mandibular Body Movement, Muscular Volumes, and Mandibular Condyle Changes

In this study, Spearman correlation revealed that changes in the amount of movement of the anterior margin of the mental foramen in the sagittal direction (*Y*-axis) had a strong positive correlation with the distance of movement and a medium positive correlation with mandibular condyle change ((R = 0.89), (*p* < 0.01) and (R = 0.50), (*p* < 0.05), respectively) in the current study. Medial pterygoid muscle volume changes had a medium positive correlation, and distance of mandibular body movement had a strong positive correlation with mandibular condyle changes ((R = 0.47), (*p* < 0.05) and (R = 0.53), (*p* < 0.01), respectively) (Table 4). Furthermore, logistic regression analysis indicated the following explanatory variables: distance of mandibular body movement and *Y*-axis directional movement of the distal segment, and changes in medial pterygoid muscle volume influenced changes in condylar morphology (outcome variable) (Table 5).

## 4. Discussion

### 4.1. Relationship between Orthognathic Surgery and TMJ Pain

The impact of orthognathic surgery on TMD (temporomandibular disorder) symptoms has been highly debated in the literature and among surgeons. Several previous studies have reported that almost 80% of symptoms were improved in patients with preexisting signs and symptoms [27,28,29]. Nevertheless, orthognathic surgery could also induce TMJ pain in those without pain before treatment [30]. Numerous studies have used two-dimensional radiographs to examine condylar position after orthognathic surgery. Other studies using 3D investigation found changes in condylar position after mandibular advancement surgery. The authors reported that the changes did not increase TMD signs and symptoms [3,4,5,6]. In addition, Harris et al. proposed that proper condylar repositioning might alleviate temporomandibular joint problems after surgery [5]. In the current study, the number of patients who presented TMJ pain was limited. Although it cannot be assessed statistically, an increase in the number of patients who presented TMJ pain following surgery was found.

### 4.2. Relationship between Orthognathic Surgery for Mandibular Advancement and Mandibular Condyle

With regard to the alterations in the condylar position that we also observed following orthognathic surgery, the condyle’s optimal intraoperative positioning was shown to sustain postoperative stability. Epker et al. recommended preserving the preoperative position of proximal mandibular segments and condyles to ensure postoperative surgical stability [7]. When less emphasis is on correcting the OJ, the condylar position may not experience significant changes. The condyles would maintain a relatively stable position within the glenoid fossa. The distal segment movement may be less pronounced than in cases with greater OJ correction. Moreover, the study of Tabrizi R et al. found that the amount of mandibular advancement in combination with the maxillary Le Fort I superior repositioning may not correlate with condylar changes; the condyles adapted approximately in their initial position nine months after the surgeries [31]. However, according to a review by Ueki et al. [8], the preoperative location of the condyle in orthognathic surgery was not the intended postoperative position. Instead, the authors suggested that the optimum position should be the position in which the remodeling volume of the TMJ generated by postoperative biomechanical stress is at its lowest, and such that no degenerative change occurs. Nevertheless, the optimum condylar position after sagittal split osteotomy is still debatable.

Morphological condyle alterations are typically found after mandibular advancement surgery [1,2,10,11,32,33,34]. Tong Xi et al. investigated 3D changes in condylar volume after surgical mandibular advancement and identified a positive correlation between decreased condylar volume and postoperative skeletal relapse [10]. Within various studies reporting on the amount of movement, a significant surgical advancement is commonly acknowledged as a risk factor for skeletal deterioration [9,34,35,36,37,38], which is consistent with the current study’s findings. Additionally, some investigations found that, in addition to the amount of surgical advancement, counterclockwise rotation of the proximal segment tended to induce mandibular condyle resorption [11,37,38] and was a predictive variable for skeletal relapse [39]. However, no significant difference was found in the current study. It seems that small rotations were observed in this study. The condyle could be within the position wherein postoperative biomechanical stress creates the least remodeling volume (physiological adaptive capability) and, thus, does not lead to morphological changes of the TMJ [8].

Some authors have studied the role of condylar torque after mandibular advancement in the development of condylar resorption [1,2,32,33,40]. Arnett et al. showed that medial or lateral torquing during orthognathic surgery could cause morphological changes in the mandibular condyle and lead to progressive condylar resorption [20], which is consistent with the present study that found a relationship between movement of the distal segment in the horizontal direction and condylar morphology. This is a possible cause governing the notion that greater the movement of the mandibular body in the horizontal direction, the more condylar torque occurs, as the procedure of fixing the gap between the proximal and distal segments during surgery requires higher force, subsequently developing resorption of the mandibular condyle. In addition, Carvalho et al., using 3D methods, observed that torque had occurred, and changes in the condylar morphology were related to the lateral movement of the mandible [40], which is also compatible with this study.

### 4.3. Relationships between Masticatory Muscle Volume Changes, Mandibular Condyle Changes, and Orthognathic Surgery

Our study observed a significant decrease in masseter and medial pterygoid muscle volumes after surgery. The results agree with Dicker et al. [16,26], who studied the adaptation of jaw-closing muscles after surgical mandibular advancement using magnetic resonance imaging (MRI). The authors found that the cross-sectional area and volume of jaw-closing muscles decreased significantly, regardless of the vertical craniofacial type. Many studies have found that the suprahyoid muscle complex, the secondary muscle of mastication, is involved in relapse following mandibular advancement surgery [9,34,35,37,41,42,43]. Due to mandibular advancement, stretching of the submandibular soft tissue and the suprahyoid muscle induces some backward displacement of the distal segment [34,43]. When the suprahyoid muscle is stretched, tension forces on the advanced mandible are directed inferiorly and posteriorly. This form of mechanical vector causes sustained pressure on the superior surface of the condyles [9,41]. A decrease in the volume of jaw-closing muscles, as demonstrated in our study and prior research by Dicker et al. [12,26], can lead to an inability to sustain these tension forces. As a result of the imbalance of forces transferred to the TMJ, the morphology of the condyle changes. Consequently, in clinical implications for surgical orthodontic treatment in skeletal class II patients, it may be worthwhile to consider including myofunctional therapy (MFT) of the jaw-closing muscles over an extended period for strengthening, in addition to the prescription of maximal mouth-opening exercises to regain normal joint function, which is usually performed for approximately two weeks following surgery [1,2,3,12]. A future study should be carried out whether extending the MFT period improves post-treatment stability.

This study has limitations, as the number of subjects involved was small. Additionally, both mono-maxillary and bimaxillary surgery were used in the study; the differences in surgical methods can influence the stability and long-term results of each procedure. Furthermore, the mandible-first approach in bimaxillary orthognathic surgery achieves more stability [44,45]. Therefore, in future research to further study the stability or whether it is different when performing mandibular setback in skeletal class III, the study should be designed with a large number of subjects, be carried out with the same surgical approach and provide dietary control for bone and muscular recovery that can affect the stability following orthognathic surgery [46]. Long-term studies would be much more valuable considering the possible changes in progress.

## 5. Conclusions

According to the present study, mandibular condyle changes were affected not only by the amount and direction of movement of the distal segment but also by the changes in the volume of the medial pterygoid muscle. These findings suggest that these factors contribute to changes in condylar morphology. These changes should be considered when planning orthognathic surgery treatment in patients with skeletal class II dentofacial deformities.

## Figures and Tables

**Table 1:** The morphological changes in the mandibular condyle.

Condylar Morphology	Pre-Treatment		Post-Treatment	
	*n*	%	*n*	%
Normal	12	20.00	4	6.67
Flattening	38	63.33	29	48.33
Erosion	5	8.33	2	3.33
Osteophytes	1	1.67	0	0.00
Flattening and erosion	4	6.67	16	26.67
Flattening and osteophytes	0	0.00	3	5.00
Flattening, erosion, and osteophytes	0	0.00	6	10.00
Total	60	100	60	100

Distribution of bone changes in accordance with the classification and their combination. *n*: number of the mandibular condyle.

**Table 2:** The positional changes in the condyle and mandibular body in three dimensions.

Measurement	Median	IQR
FH Condyle angle (degree)	−2.56	−0.51–(−4.36)
MSR Condyle angle (degree)	0.85	3.56–(−1.83)
N Condyle angle (degree)	−0.86	1.28–(−1.46)
Distance (mm)	5.67	7.69–3.84
Direction (mm) *X*	0.61	1.61–(−0.44)
*Y*	4.67	7.18–2.85
*Z*	0.23	1.99–(−1.30)

IQR: interquartile range.

**Table 3:** The changes in the masseter and medial pterygoid muscle volumes.

Measurement	Pre-Surgery		Post-Surgery		*r*	*p* Value
	Median	IQR	Median	IQR		
Masseter (cm^3^)	16.96	20.38–13.75	15.70	17.76–12.82	0.60	0.00 **
Medial pterygoid (cm^3^)	7.31	9.16–6.30	6.56	7.86–5.90	0.62	0.00 **

Wilcoxon signed-rank test; **: *p* < 0.01: Statistically significant difference; IQR: interquartile range.

**Table 4:** Correlations between changes in the position of the condyle, movement of the mandibular body, muscular volume, and condylar morphology changes.

Variables	Correlation		Variables	Correlation	
	R	*p* Value		R	*p* Value
MCA vs. masseter	−0.15	NS	*X* axis vs. masseter	0.05	NS
MCA vs. M ptery	−0.17	NS	*X* axis vs. M ptery	0.04	NS
MCA vs. condyle	0.01	NS	*X* axis vs. condyle	−0.07	NS
MCA vs. distance	−0.07	NS	*X* axis vs. distance	0.18	NS
FCA vs. masseter	0.08	NS	*Y* axis vs. masseter	0.25	NS
FCA vs. M ptery	0.33	NS	*Y* axis vs. M ptery	0.07	NS
FCA vs. condyle	0.09	NS	*Y* axis vs. condyle	0.50	0.01 *
FCA vs. distance	−0.04	NS	*Y* axis vs. distance	0.89	0.00 **
NCA vs. masseter	−0.02	NS	*Z* axis vs. masseter	−0.10	NS
NCA vs. M ptery	−0.02	NS	*Z* axis vs. M ptery	0.08	NS
NCA vs. condyle	−0.06	NS	*Z* axis vs. condyle	0.18	NS
NCA vs. distance	0.22	NS	*Z* axis vs. distance	0.12	NS
condyle vs. masseter	0.29	NS	masseter vs. M ptery	0.29	NS
condyle vs. M ptery	0.47	0.01 *	masseter vs. distance	0.23	NS
condyle vs. distance	0.53	0.00 **	M ptery vs. distance	0.15	NS

Spearman rank correlation; *: *p* < 0.05, **: *p* < 0.01: Statistically significant difference; NS: Not significantly different; M ptery: Medial pterygoid; masseter: Masseter; MCA: midsagittal plane-condyle angle; FCA: Frankfort horizontal plane-condyle angle; NCA: nasion planecondyle angle; condyle: Condylar morphology changes; distance: distance of mandibular body movement; *X* axis: positional changes in the *X*-axis direction; *Y* axis: positional changes in the *Y*-axis direction; and *Z* axis: positional changes in the *Z*-axis direction.

**Table 5:** Evaluations of the risk for morphological changes in the mandibular condyle.

Evaluation		B	SE	Expo (B)	95% Cl		R^2^	*p* Value
					Lower	Upper		
Condyle angle	MCA	0.01	0.06	1.01	0.89	1.13	0.00	0.91
	FCA	0.08	0.12	1.89	0.87	1.37	0.01	0.47
	NCA	−0.02	0.18	0.98	0.69	1.40	0.00	0.91
Mandibular body movement								
Distance		0.75	0.31	2.12	1.17	3.85	0.30	0.01 *
Direction	*X*	0.02	0.20	1.02	0.68	1.51	0.05	0.93
	*Y*	0.45	0.22	1.57	1.03	2.39	0.25	0.04 *
	*Z*	0.22	0.20	1.24	0.84	1.84	0.03	0.28
Masseter volume change		0.43	0.28	1.53	0.89	2.63	0.07	0.12
Medial pterygoid volume change		2.04	0.97	7.66	1.14	51.37	0.22	0.04 *

*: *p* < 0.05: Statistically significant difference.

## Data Availability

The datasets used and analyzed during the current study are available from the corresponding author upon reasonable request.
